# Inhibitory Effect of Ursolic Acid on the Migration and Invasion of Doxorubicin-Resistant Breast Cancer

**DOI:** 10.3390/molecules27041282

**Published:** 2022-02-14

**Authors:** Li Zong, Guorong Cheng, Jingwu Zhao, Xiaoyu Zhuang, Zhong Zheng, Zhiqiang Liu, Fengrui Song

**Affiliations:** 1 State Key Laboratory of Electroanalytical Chemistry, Changchun Institute of Applied Chemistry, Chinese Academy of Sciences, Changchun 130022, China; zongliciac@126.com (L.Z.); mslab26@ciac.ac.cn (G.C.); mslab24@ciac.ac.cn (J.Z.); zhengzh@ciac.ac.cn (Z.Z.); liuzq@ciac.ac.cn (Z.L.); 2 Jilin Province Key Laboratory of Chinese Medicine Chemistry and Mass Spectrometry, Changchun Institute of Applied Chemistry, Chinese Academy of Sciences, Changchun 130022, China; 3 Experiment Center for Science and Technology, Shanghai University of Traditional Chinese Medicine, Shanghai 201203, China

**Keywords:** ursolic acid, metastasis, multidrug resistance, polyamine metabolism, ornithine decarboxylase

## Abstract

The cause of death in most breast cancer patients is disease metastasis and the occurrence of multidrug resistance (MDR). Ornithine decarboxylase (ODC), which is involved into multiple pathways, is closely related to carcinogenesis and development. Ursolic acid (UA), a natural triterpenoid compound, has been shown to reverse the MDR characteristics of tumor cells. However, the effect of UA on the invasion and metastasis of tumor cells with MDR is not known. Therefore, we investigated the effects of UA on invasion and metastasis, ODC-related polyamine metabolism, and MAPK-Erk-VEGF/MMP-9 signaling pathways in a doxorubicin-resistant breast cancer cell (MCF-7/ADR) model. The obtained results showed that UA significantly inhibited the adhesion and migration of MCF-7/ADR cells, and had higher affinities with key active cavity residues of ODC compared to the known inhibitor di-fluoro-methyl-ornithine (DFMO). UA could downregulate ODC, phosphorylated Erk (P-Erk), VEGF, and matrix metalloproteinase-9 (MMP-9) activity. Meanwhile, UA significantly reduced the content of metabolites of the polyamine metabolism. Furthermore, UA increased the intracellular accumulation of Dox in MCF-7/ADR cells. Taken together, UA can inhibit against tumor progression during the treatment of breast cancer with Dox, and possibly modulate the Erk-VEGF/MMP-9 signaling pathways and polyamine metabolism by targeting ODC to exert these effects.

## 1. Introduction

Breast cancer has the highest incidence and mortality rate among women worldwide, with an estimated 2,261,419 new cases and 684,996 deaths in 2020 according to the data obtained from the GLOBOCAN 2020 online database [1]. Two major obstacles in breast cancer treatment are therapeutic resistance and the formation of metastasis to secondary sites (lung, bone, lymph nodes, brain, and liver) [2]. The emergence of multidrug resistance (MDR) severely reduces the efficacy of antitumor drugs, further leading to a decline in survival rate. Moreover, MDR is also closely related with rapid recurrence and metastasis in cancer patients. Tumor cells with MDR exhibit enhanced invasion and migration abilities compared with drug-sensitive cells [3]. Increased attention has been focused on the finding of drugs with high activity and low toxicity for targeting tumor MDR, metastasis and recurrence. In this regard, phytocompounds, proven as a powerful chemopreventive and chemotherapeutic strategy in various cancers, including breast cancer, could be considered as a powerful resource for finding potential anticancer drugs [4]. Targeting polyamine metabolism has been suggested to be a promising strategy for overcoming drug resistance. Moreover, polyamine metabolism is associated with breast cancer metastasis.

Polyamines (mainly putrescine, spermidine, and spermine) are a class of amines that is protonated at physiological pH, and thus binds to and regulates negatively charged DNA, RNA, protein, and phospholipids by electrostatic interaction [5]. Therefore, an increase in polyamines is closely associated with enhanced abilities for cell proliferation, invasion, and metastasis [6]. Increased levels of polyamines have been found in many solid tumors and have been used as important biomarkers in tumor diagnosis, treatment, and prognosis [5]. Polyamine levels are highly regulated by ornithine decarboxylase (ODC), which is an important rate-limiting enzyme in the polyamine metabolism pathways and can catalyze the conversion of ornithine (Orn) into putrescine (Put). Enhanced ODC activity has been found in a variety of tumors, and α-difluoromethylornithine (DFMO), an irreversible inhibitor of ODC, can inhibit tumor proliferation and metastasis.

ODC regulated invasion and metastasis ability by multiple signaling pathways. The inhibition of ODC suppresses the development of esophageal precancerous lesions by downregulating p38 α and Erk1/2 in the mitogen-activated protein kinase (MAPK) pathway and AKT/mTOR/p70S6k signaling pathways [7]. The overexpression of ODC and COX-2 together leads to the pathogenesis of aggressive and invasive cutaneous carcinomas by regulating the Akt-Erk signaling pathway [8]. Elevated levels of ODC can cooperate with Raf/Erk activation to convert normal keratinocytes into invasive malignant cells [9]. The activated Src/Mek/Erk/c-Myc pathway can upregulate ODC expression [10]. Polyamine metabolism can also be regulated by the Ras-Raf-Mek-Erk signaling pathway in multiple aspects [11]. Therefore, the expression and action of ODC are closely related to the activity of the Erk kinase, which is a central element of the MAPK signaling pathways involved in various cell activities such as cell survival, dissemination, and multidrug resistance (MDR) [12].

During tumor progression, vascular endothelial growth factor (VEGF) and matrix metalloproteinases (MMPs) play vital roles [13]. It is reported that a positive feedback loop might exist between the Erk1/2 and VEGF signaling pathways. In addition, Erk signals also regulate MMP-9 to promote growth, invasion, angiogenesis, and metastasis [13]. Therefore, ODC is possibly implicated in tumor progress through the Erk1/2 signaling and VEGF signaling pathways.

Ursolic acid (UA) is a naturally occurring pentacyclic triterpenoid compound that is widely found in many foods and herbs, such as apple, cranberry, and rosemary. It has a variety of biological activities, including anti-inflammatory, antiviral, antibacterial, antidiabetic, and immune-modulatory effects, especially antitumor activity [14]. The anticancer mechanisms of UA occur mainly via modulating multiple tumor-related signaling pathways, such as PI3K/Akt/mTOR-, Erk- and EGFR-related signaling cascades [4]. UA also caused the significant reversal of the MDR in MCF-7/ADR cells in our previous work [15]. In addition, UA also shows inhibitory effects on the invasion and metastasis of human cancer cells in vitro and in vivo, and is a potential anti-metastatic agent [16]. However, the inhibitory effect of UA on the invasion and metastasis of tumor cells with MDR is not known.

Therefore, the inhibition of UA on the invasion and metastasis of doxorubicin-resistant MCF-7/ADR cells was investigated in this work. In addition, to explore the underlying molecular mechanism of UA in the invasion and metastasis of breast cancer cells with MDR, we also studied the expression levels of ODC, MMPs, MAPK/Erk, and VEGF signaling proteins, and the metabolic pathways of polyamine.

## 2. Results

### 2.1. UA Reduced Adhesion of MCF-7/ADR Cells to HUVEC Cells

**Figure 1:**
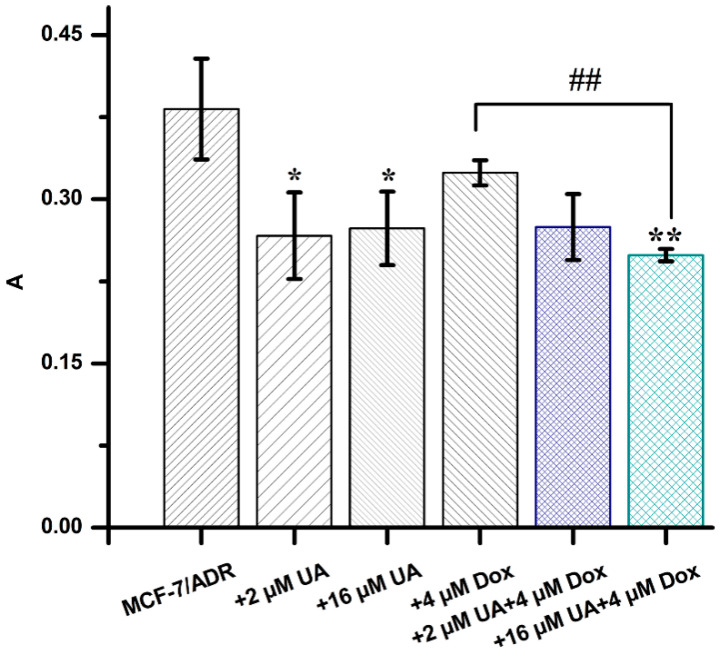
The absorbance values of MCF-7/ADR cells treated with different drug combinations, obtained from the cell adhesion assay. * *p* < 0.05, ** *p* < 0.01 compared with MCF-7/ADR cells, ## *p* < 0.01 vs. MCF-7/ADR cells treated with 4 µM Dox.

The adhesion of tumor cells to vascular endothelial cells is the key step in the invasion of tumor cells, so effectively inhibiting heterogeneous adhesion is a powerful pathway to prevent tumor metastasis. In this study, the heterogeneous adhesion of tumor cells to HUVEC cells was observed by the dye Rose bengal. It was clearly shown that the amount of MCF-7/ADR cells adhering to HUVEC cells was significantly reduced after 48 h of treatment with UA or the combination of UA and Dox (Figure 1). It was found that 4 µM Dox had no significant effect on the adhesion of MCF-7/ADR cells to HUVEC cells compared with the control group, which may be attributed to the occurrence of Dox resistance in the MCF-7 cells, and the fact that chemotherapy can promote the tumor metastasis [17,18]. MCF-7/ADR cells were established by exposing parental MCF-7 cells to increasing concentrations of adriamycin (from 0.01 to 1 μg/mL) and then maintained in medium containing 1 μg/mL adriamycin for two months [19]. Therefore, MCF-7/ADR cells stimulated with Dox should produce a similar response to that of MCF-7/ADR cells. Moreover, the combination of 16 µM UA and 4 µM Dox inhibited adhesion significantly compared with the control group (*p* < 0.01) and treatment group by 4 µM Dox alone (*p* < 0.05). These data indicated that UA could reduce the adhesion ability of MCF-7/ADR, with an obvious inhibitory effect for the combination of UA and the chemotherapeutic drug Dox.

### 2.2. UA Inhibited Migration of MCF-7/ADR Cells

**Figure 2:**
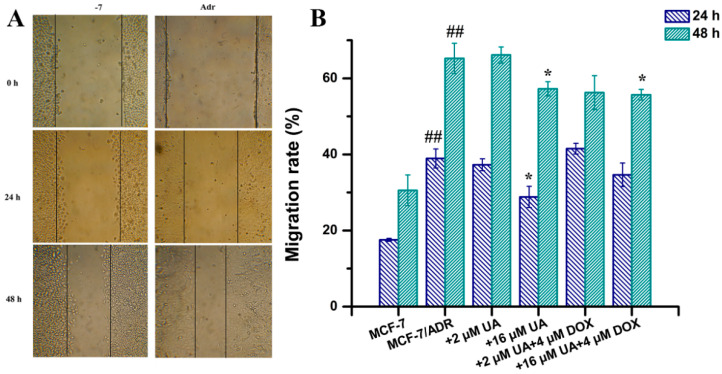
The effects of drugs in different combination on cell migration ability. (**A**): representative images from the wound healing assay. The cells forming a confluent monolayer were scratched by a 1 mL pipette tip to create an incision-like gap and then photographed at defined time points. (**B**): migration rates calculated by measuring the distance of the gap at different time points, to quantify the cell migration ability determined from the wound healing assays. * *p* < 0.05, compared with MCF-7/ADR cells, ## *p* < 0.01 compared with MCF-7 cells.

The migration ability of tumor cells is indispensable for tumor metastasis. The effect of UA on the migration ability of MCF-7/ADR cells was investigated by wound-healing assay and compared with drug-sensitive MCF-7 cells. The results showed that the migration distance of MCF-7/ADR cells was greater compared with that of MCF-7 cells, indicating that the enhanced migration ability of MCF-7 was due to the occurrence of MDR (Figure 2A). The migration rate of MCF-7/ADR cells was significantly reduced after 16 μM UA treatment for 24 h and 48 h. Moreover, the migration rate of MCF-7/ADR cells treated with 16 μM UA combined with 4 μM Dox for 48 h was also significantly reduced compared with that of MCF-7/ADR cells (Figure 2B). The migration rate of MCF-7/ADR cells was also decreased after treatment with the combination of 2 μM UA and 4 μM Dox for 48 h, although there was no significant difference from the control group.

### 2.3. UA Reduced the Infiltration Ability of MCF-7/ADR Cells to HUVEC Cells

The infiltration of tumor cells into vascular endothelial cells is a prerequisite for tumor metastasis. In order to closely mimic solid tumors, we established a multicellular tumor spheroids (MCTs) model. Three-dimensional MCTs have the characteristics of tumor heterogeneity, hypoxia, and nutrient gradients, and have been an in vitro model for cancer biology research [20]. An infiltration model of MCF-7/ADR MCTs into HUVEC cells was established to better simulate the in vivo process of tumor metastasis.

**Figure 3:**
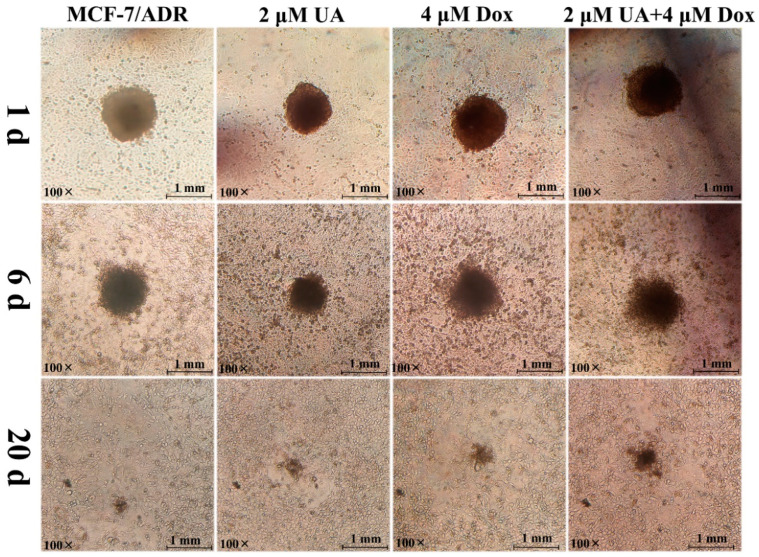
Representative images of infiltration of multicellular tumor spheroids incubated with different drug combinations on the HUVEC monolayer cells at 1 d, 6 d, and 20 d.

After co-culture of MCF-7/ADR MCTs and HUVEC cells for 1 day, MCF-7/ADR MCTs were observed to deposit onto the HUVEC monolayers, but MCTs would float with slight shaking. The compactness of MCF-7/ADR MCTs and the invasion area changed over time. On the 6th day, MCF-7/ADR MCTs continued to infiltrate the HUVEC monolayer, dispersed widely, and replaced the HUVEC cells. Until the 20th day, the MCTs dissociated completely and the MCT cells and HUVEC monolayer cells were in the same plane. However, the dissociation rate and infiltration area of MCF-7/ADR MCTs treated with UA alone or in combination with Dox were reduced, respectively, and HUVEC retraction also was reduced (Figure 3). These results indicated that UA could increase the homogenous adhesion of tumor cells, reduce the heterogeneous adhesion of tumor cells to other cells, and thus inhibit tumor cell invasion.

### 2.4. UA Had a Higher Binding Affinity for ODC Protein Compared with DFMO

Ornithine decarboxylase (ODC) is one of the most important rate-limiting enzymes in polyamine biosynthesis. The depletion of polyamines in tumor cells can inhibit proliferation, invasion, and metastasis, and the production of polyamines in vivo could be inhibited by suppressing the activity of ODC. The interaction of UA and ODC was studied by molecular docking analysis.

**Figure 4:**
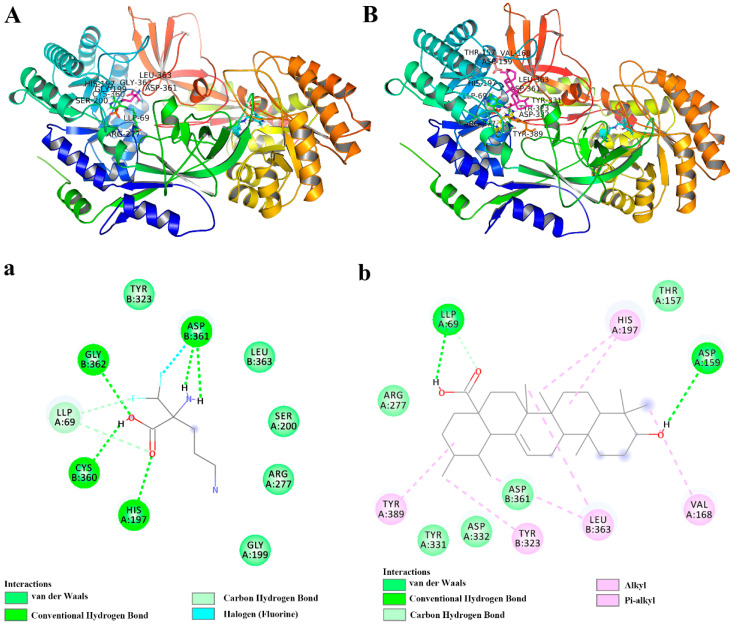
The docking analysis of protein and ligand interactions. The 3D interaction model of ODC protein and known inhibitor DFMO (**A**) and UA (**B**). The 2D diagram showing interactions of ODC protein and known inhibitor DFMO (**a**) and UA (**b**).

Initially, the docking of known inhibitor DFMO with ODC was performed as an internal control. The results showed that DFMO formed hydrogen bonds with the residues LLP69, His197A, Cys360B, Asp361B, and Gly362B, and van der Waals forces between the residues Gly199A, Ser200A, Tyr323B, Leu363B, coincident with the reported literature (Figure 4a) [21]. Moreover, Cys360 and pyridoxal 5-phosphate (PLP, LLP69) play a major role in interacting with ligands, thus inhibiting ODC. The results also indicated that the adopted method was successful.

Similarly, the interaction analysis of UA and ODC showed that UA formed hydrogen bonds with the residues LLP69A and Asp159A, and van der Waals forces between the residues Thr157A, Arg277A, Tyr331A, Asp332A, Asp361B. Furthermore, UA, a pentacyclic triterpenoid, could form hydrophobic interactions, including π–alkyl and alkyl–alkyl interactions, with the residues Val168A, His197A, Tyr389A, Tyr323B, and Leu363B (Figure 4b). Moreover, it is reported that DFMO could stabilize the hydrophobic cavity encompassing the Asp332A, Asp362, Gly362B, Leu363B, Tyr323B, Tyr331A, Tyr389A, and Phe397B residues [21], consistent with our obtained data, and these also indicated that DFMO and UA had similar binding sites (Figure 4A,B). The binding energy of DFMO and UA with ODC was calculated as −3.94 and −5.25 kcal/mol, respectively, indicating that UA had a higher affinity for the active catalytic sites of ODC than DFMO. While the clinical use of DFMO is limited due to the high dose and severe side effects, UA, a natural compound, has been evidenced to have many biological activities at low doses. Therefore, our results indicated that UA is a potential agent for targeting ODC, although further research is needed to confirm the important role of ODC in the inhibitory effect of UA on metastasis.

### 2.5. UA Inhibited ODC, VEGF, MMP-9 Activity and Erk1/2 Phosphorylation in MCF-7/ADR Cells

**Figure 5:**
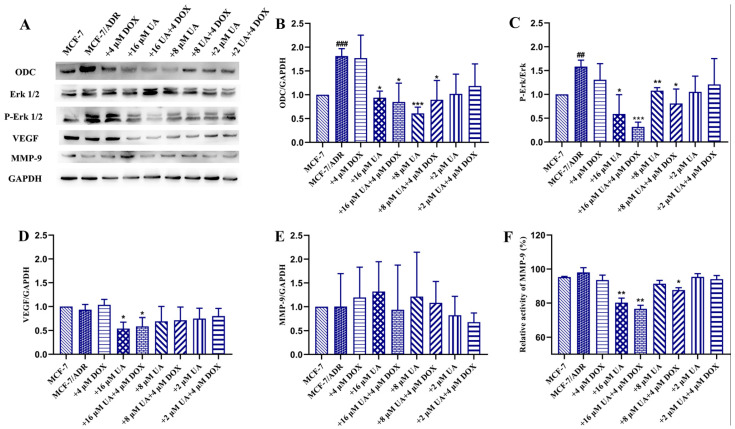
The effects of different concentrations of UA alone or in combination with Dox on ODC and phosphorylation of Erk1/2, VEGF, MMP-9. (**A**): representative immunoreactive bands of ODC, VEGF, MMP-9, GAPDH, total and phosphorylated Erk1/2 using specific antibody. (**B**–**E**): representative quantification of ODC, phosphorylated Erk1/2, VEGF, MMP-9. (**F**): determination of MMP-9 activity. * *p* < 0.05, ** *p* < 0.01, *** *p* < 0.001 compared with MCF-7/ADR cells, ## *p* < 0.01, ### *p* < 0.001, comparison between MCF-7/ADR group and MCF-7 group.

ODC is related to tumor progression via multiple signaling pathways, especially the Erk-related signaling pathways for tumor invasion and metastasis. Therefore, further investigation of Erk-related signaling pathways was performed. As can be seen from Figure 5, there was no significant difference between the MCF-7/ADR cells treated with Dox alone and the control cells. However, UA could significantly inhibit the expression of ODC in MCF-7/ADR cells and the expression of ODC in drug-resistant cells was obviously higher than that in drug-sensitive cells, consistent with the literature report [22]. Moreover, the combination of UA and Dox could inhibit the expression of ODC in MCF-7/ADR cells, although no significant difference was observed with 2 μM UA alone or in combination with 4 μM Dox compared to the MCF-7/ADR group.

In addition, ODC could affect angiogenesis and the MAPK pathway, thus affecting the production of metalloproteinases [23]. It is found that the phosphorylation of Erk1/2 and the expression of angiogenic factors, such as VEGF, were increased in MCF-7/ADR cells compared to the MCF-7 cells, and inhibited after treatment with UA. The combination of UA and Dox exhibited a more obvious effect on inhibition of the phosphorylation of Erk1/2 and VEGF expression, especially the combination of 16 μM UA and 4 μM Dox, indicating that UA could decrease the increased VEGF levels induced by Dox. The obtained results coincided with the literature report that enhanced expression of VEGF receptor 1 promoted tumor metastasis induced by chemotherapy [24]. Moreover, UA significantly inhibited the activity of MMP-9, but no obvious inhibition of its expression was observed. The combined effect of UA and Dox on inhibition of MMP-9 activity was pronounced. The impact of UA on MMP-2 expression was inconclusive because an extremely low level of MMP-2 is expressed in MCF-7/ADR cells, even in the absence of UA (data not shown). These data indicated that the signaling pathways involving ODC, Erk1/2, VEGF, and MMP were related to the MDR, as shown by comparing MCF-7 cells and MCF-7/ADR cells, and the action of UA was shown by comparing MCF-7/ADR cells treated by UA and control cells.

### 2.6. UA Inhibited Polyamine Metabolism in MCF-7/ADR Cells

**Figure 6:**
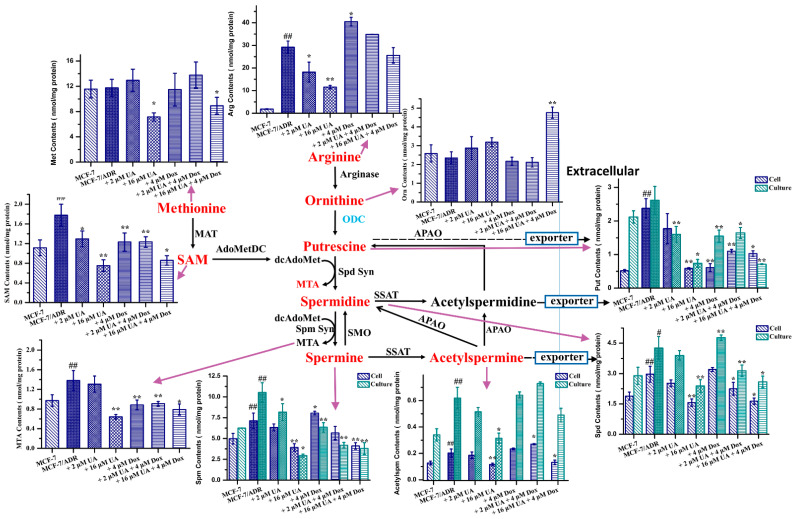
The quantitative analysis of metabolites involved into the polyamine metabolic pathways. SAM, S-adenosylmethionine; MTA, 5’-methylthioadenosine; Put, putrescine; Spd, spermidine; Spm, spermine; Aceytlspm, N-acetylspermine; Orn, ornithine; Arg, arginine; Met, methionine. * *p* < 0.05, ** *p* < 0.01 compared with MCF-7/ADR cells, # *p* < 0.05, ## *p* < 0.01 compared with MCF-7 cells.

As ODC is correlated with polyamine metabolism, the small-molecule endogenous metabolites involved in the polyamine metabolic pathways in tumor cells were quantified by UPLC–MS/MS (Figure 6). The results show that significant changes in metabolites were present between MCF-7 and MCF-7/ADR cells; the levels of most metabolites were significantly increased in MCF-7/ADR cells compared with MCF-7 cells, although methionine was not significant (*p* > 0.05), consistent with the reported literature [25]. This also indicated that polyamine metabolism played an important role in the occurrence and development of MDR. Most of these metabolites associated with polyamine metabolism were decreased in MCF-7/ADR cells treated with UA for 48 h in addition to the significant increase in ornithine. Moreover, the effect was greater than that of Dox alone. The intracellular and extracellular contents of Put, Spd, Spm, and Acetylspm were decreased after the treatment with the combination of UA and Dox. These data suggested that UA could alter polyamine metabolism in MCF-7/ADR cells, consistent with the decreased expression of ODC.

### 2.7. UA Increased the Intracellular Accumulation of Dox in MCF-7/ADR Cells

**Figure 7:**
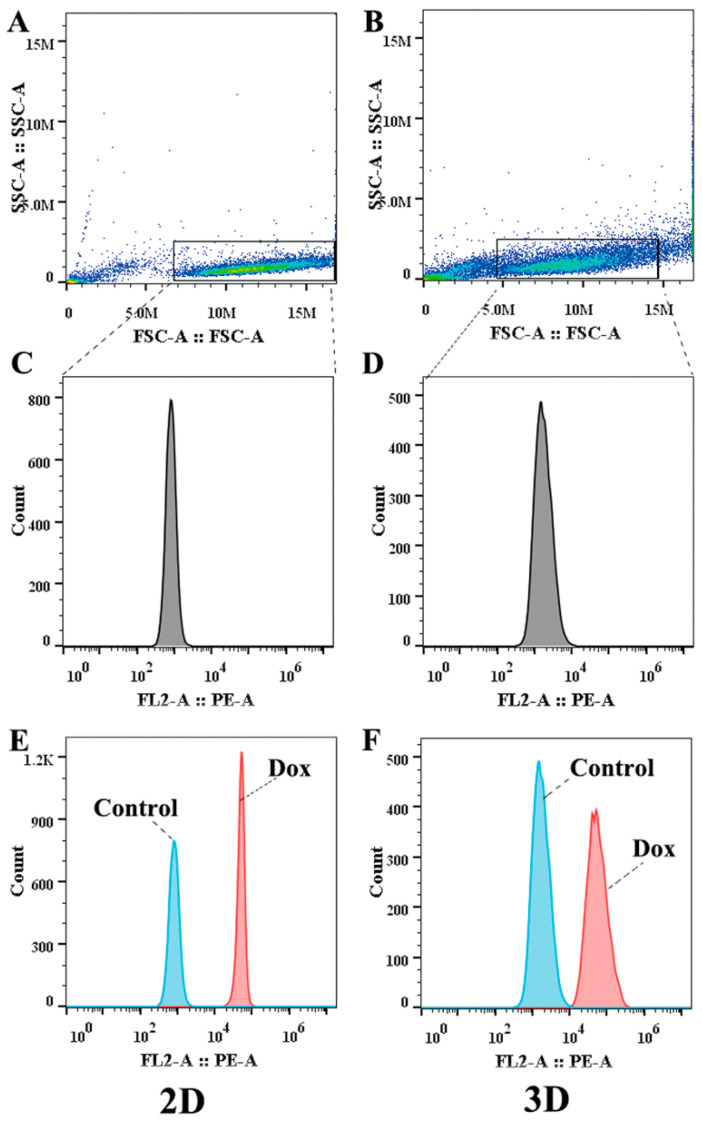
The results measured by flow cytometry. (**A**,**B**) are the scattergrams of cells cultured in 2D and 3D. (**C**,**D**) are the histograms of gated cells cultured in 2D and 3D. (**E**,**F**) show accumulation of Dox in 2D and 3D cells.

It was revealed that cells in 3D MCTs had significantly enhanced resistance to chemotherapy and enhanced invasion and metastasis abilities compared to conventional monolayer cells [26]. Therefore, the MCF-7/ADR MCTs model was established to evaluate the effect of UA on the intracellular accumulation of Dox in spheroid cells and monolayer-cultured cells were used for comparative analysis. Considering the presence of MDR in MCTs and the sensitivity of the instrument, we investigated the effect of different concentrations of UA on the intracellular accumulation of 10 μM Dox. It was found that the accumulation of Dox in spheroid cells was lower than that in the cultured monolayer, indicating that the cells in the MCTs differed from the cultured-monolayer cells. The combination of Dox with different concentrations of UA could still increase the Dox content of cells in the MCTs, although there was no significant difference compared with the control group (*p* > 0.05, Table 1). The more dispersed distribution in the scatter plot obtained by flow cytometry also demonstrated the heterogeneity of MCF-7/ADR cells present in MCTs (Figure 7).

## 3. Discussion

The occurrence of tumor MDR and the invasion and metastasis to other organs are the main causes leading to the death of breast cancer patients. As mentioned above, ODC is involved into multi-signaling pathways, and thus affects the development and progress of cancer invasion and metastasis. UA, a natural compound, has been shown to reverse MDR [15]. ODC is relevant to Erk-related signaling pathways and polyamine metabolism pathways [7,8,9,10], and VEGF and MMPs have a key role in cancer invasion and metastasis [13]. Therefore, the effect of UA on the cancer invasion and metastasis was investigated and the underlying molecular mechanisms were also further explored.

It is reported that chemotherapy could induce the enhanced metastasis ability of tumor cells. Our results confirmed that UA alone or in combination with the chemotherapy drug Dox could inhibit the adhesion, infiltration, and metastasis ability of breast cancer cells with MDR. Furthermore, UA also slightly increased the intracellular accumulation of Dox in a three-dimensional MCF-7/ADR cell model, which enhanced the cytotoxic effect of Dox.

The interaction of tumor cells with vascular endothelial cells is a critical stage for distant metastasis, which is involved in the adhesion to the vascular endothelium and extravasation or invasion of the vascular endothelium and underlying basement membrane [27]. MMP-9 secreted by invasive cancer cells can degrade the proteins in the extracellular matrix (ECM), which further destroys the histological barriers to tumor cell invasion, and plays a key role in tumor invasion and metastasis [28]. Our results showed that UA alone or in combination with Dox inhibited the activity of MMP-9. VEGF, an angiogenic factor, can promote the expression of MMP-9. UA alone or in combination with Dox inhibited the expression of VEGF, which has been confirmed to be closely related to angiogenesis, drug resistance, and tumor growth [29].

The Erk1/2 signaling pathways are associated with ODC in addition to VEGF and MMPs. ODC is the first rate-limiting enzyme in polyamine synthesis, and is upregulated in breast cancer [5]. The inhibition of both ODC and S-adenosylmethionine decarboxylase 1 leads to polyamine depletion and tumor suppression. The expression and activity of ODC have been considered as prognostic markers. Moreover, ODC was implicated in the regulation of estrogen receptor α expression. In estrogen receptor α-positive MCF-7 and T-47D breast cancer cells, ODC knockdown could diminish the mRNA and protein expression of estrogen receptor α [30]. On this basis, ODC plays a vital role in the tumorigenesis and progression of breast cancer. Furthermore, many studies have shown that polyamines are involved in the expression and activation of mitogen-activated protein kinase (MAPK), and the activity of MAPK in ODC-overexpressing transfected cells is significantly enhanced [31]. Our results showed that UA, alone or in combination with Dox, suppressed the expression of ODC. Molecular docking also confirmed that UA had a higher affinity to ODC, which could further affect ODC activity. Based on the obtained results, it can be deduced that UA could target ODC to exert its effect against invasion and metastasis in breast cancer. Besides, UA alone or in combination with Dox can inhibit the phosphorylation of Erk1/2, further influencing the downstream signaling molecules, as evidenced by the decreased activity of MMP-9 and expression of VEGF.

Besides, ODC-related polyamine metabolism has been revealed to be associated with tumor MDR, invasion, and metastasis [32]. Therefore, we conducted an analysis of polyamine-related metabolites.

The starting point of polyamine metabolism is ornithine, and ornithine is the product of arginine catalyzed by arginase; thus, arginine (Arg) is closely related to the polyamine metabolic pathways [33]. Although Arg is a non-essential amino acid, it is involved in protein synthesis, cell cycle regulation, and other cellular activities. Moreover, increased demand was observed under some pathological conditions, such as tumors, inflammation, and organ dysfunction, and as the Arg synthesized by the urea cycle did not meet the increased demand exogenous Arg was a main source. Consequently, Arg is also called a semi-essential amino acid [34,35]. The obtained results showed that Arg levels in multidrug-resistant MCF-7/ADR cells were higher than in the drug-sensitive MCF-7 cells, indicating that MCF-7/ADR cells had a high demand for the Arg. The intracellular levels were reduced after treatment with UA or the combination of UA and Dox, which indicated that UA could regulate arginine metabolism.

The conversion of ornithine to putrescine is catalyzed by ODC. The level of ornithine in MCF-7/ADR cells was not significantly different from that in MCF-7 cells, while the levels of ornithine in MCF-7/ADR cells were increased after the treatment of UA or UA combined with Dox. This could be ascribed to a decrease in ODC expression and the inhibition of ODC activity, evidenced by the western blot and molecular docking.

Methionine (Met) is involved into the conversion of putrescine (Put) to spermidine (Spd) and of spermidine to spermine (Spm), during which decarboxylated S-adenosylmethionine (dcAdoMet) is converted to 5’-methylthioadenosine (MTA). The obtained results for Met quantification showed that the content of Met in MCF-7/ADR cells was higher than that in MCF-7 cells, although there was no significant difference, and high concentrations of UA alone or in combination with Dox could reduce the content of Met. The results coincided with the previous research that reducing the Met content could increase the efficacy of chemotherapeutic drugs and inhibit the migration of tumor cells [36].

MTA not only participates the polyamine synthesis, but also is a starting point of the purine salvage pathway. It plays a regulatory role in cell functions, including gene expression, cell proliferation, lymphocyte activation, apoptosis, and even tumor development and invasiveness [37]. The increased levels in tumor cells may be ascribed to the lower level of MTA phosphorylase (MTAP) or the loss of MTAP activity, which converts MTA into 5-methylthioribose-1-phosphateandadenine [38]. MTA’s role in tumor cell proliferation, migration, and invasion is controversial [39,40]. In this study, we found that the levels of MTA increased in MCF-7/ADR cells and decreased after treatment with UA, consistent with the reported literature [39]. Moreover, UA combined with Dox could reduce the levels of MTA and the inhibitory effect was better than that of Dox alone.

S-adenosylmethionine (SAM) is the product of Met by the action of methionine adenosyltransferase (MAT), and is an important methyl donor for the methylation of intracellular nucleic acids, proteins, and other biomacromolecules [41]. It was detected that the SAM content in MCF-7/ADR cells was higher than that in MCF-7 cells, indicating that methylation could be related to the occurrence and development of MDR. Moreover, UA alone or in combination with Dox could reduce the SAM content and the inhibitory effect was better than that of Dox alone. The decreased SAM content could be caused by the decreased upstream Met content.

The acetylation of polyamines is important for maintaining the homeostasis of intracellular polyamines via removing excess intracellular polyamines from the cells, thereby maintaining the intracellular polyamine content within a reasonable range as required by the cells [42]. The increased level of extracellular acetylspermine may be associated with an increased rate of polyamine catabolism, meanwhile also indicating a high rate of polyamine synthesis. UA alone or in combination with Dox could decrease the levels of intracellular and extracellular acetylspermine, and the inhibitory effect was better than that of Dox alone, indicating that the polyamine metabolism was blocked by UA in MCF-7/ADR cells and as a consequence of the decreased levels of upstream metabolites.

In summary, the obtained results from the quantification of polyamine metabolite showed that UA alone or combined with Dox could inhibit polyamine metabolism, and the effects were better than Dox alone (Figure 6). These were beneficial for inhibiting tumor progression, because polyamines could induce tumor progression as mentioned above. The results of molecular docking and western blotting showed that the ODC levels and activity decreased after the treatment with UA alone or combined with Dox. ODC is the first rate-limiting enzyme in polyamine metabolism. Therefore, it can be deduced that this was a consequence of ODC inhibition.

## 4. Materials and Methods

### 4.1. Materials

UA with a purity of above 98% was purchased from Shanghai Winherb Medical Science Co., Ltd. (Shanghai, China). Doxorubicin (Dox) with a purity of above 99% was purchased from Dalian Meilun Biotechnology Co., Ltd. (Dalian, China). HPLC-grade acetonitrile and formic acid were purchased from Fisher Scientific (Loughborough, UK). Ultrapure water was prepared using a Milli-Q plus (Milford, MA, USA). Putrescine (Put), spermidine (Spd), spermine (Spm), N-acetylspermine (Aceytlspm), ornithine (Orn), arginine (Arg), methionine (Met), S-adenosylmethionine (SAM), and 5’-methylthioadenosine (MTA) were purchased from Sigma-Aldrich (St. Louis, MO, USA). The internal standard 1,6-diaminohexane was purchased from Aladdin (Shanghai, China). Antibodies against MMP-9, VEGF, ODC, GAPDH, and total and phosphorylated Erk1/2 were purchased from Affinity Biosciences (Oklahoma, USA). The assay kit for MMP-9 activity was purchased from Shanghai Jianglai Biotechnology Co., Ltd. (Shanghai, China). Breast cancer cell lines MCF-7/ADR and MCF-7 were purchased from Peking Union Medical College Cell Resource Center (Beijing, China). The HUVEC cell line was purchased from Shanghai Bogu Biotechnology Co., Ltd. (Shanghai, China).

### 4.2. Cell Culture

MCF-7/ADR and MCF-7 cells were cultured according to previous work [19], and the culture method for HUVECs was same as that for MCF-7 cells.

### 4.3. Cell Adhesion Assay

An assay of the ability of MCF-7/ADR cells to adhere to HUVECs was used to investigate the effect of drugs on the adhesion ability of MDR cells. HUVECs were seeded in 96-well plates at 8.5 × 10^4^ cells/well, and achieved 100% confluence after 72 h culture. Then, the HUVECs were washed with cell culture medium, and 200 μL of 5 × 10^5^/mL MCF-7/ADR cell suspension treated with 4 μM Dox, 2 μM UA, 16 μM UA, and different concentrations of UA in combination with 4 μM Dox for 48 h was added to each well. There were 6 parallel wells per group. After co-culture for 2 h, the cell culture medium was discarded, and the non-adherent cells were washed away with the cell culture medium. Then, 100 μL of 0.25% Rose bengal solution in phosphate-buffered saline (PBS; pH 7.2, 0.01 M) was added and the cells were stained for 5 min in the cell culture incubator. After washing with cell culture medium, 200 μL of 95% ethanol:PBS (1:1) solution was added to each well, allowed to stand at room temperature for 1 h, and the absorbance at 570 nm was measured by a microplate reader. The adhesion ability was calculated as follow: absorbance value of tumor cells = absorbance value of tumor cells and endothelial cells − absorbance value of endothelial cells.

### 4.4. Cell Migration Assay

The effect of UA on the migration ability of MDR cells was determined by the wound healing assay. Three horizontal lines were evenly drawn at the bottom of the 6-well plate with the black marker. MCF-7/ADR and MCF-7 cells (4 × 10^5^ cells per well) were seeded in the 6-well plates; the corresponding drugs were added after overnight adhesion (2 μM UA, 16 μM UA, 4 μM Dox+2 μM UA, and 4 μM Dox+16 μM UA) and cultured for 48 h to reach 100% confluence. There were 3 parallel wells in each group. Then, 3 lines perpendicular to the horizontal line were carefully drawn with a 1 mL pipette tip. The cells were washed twice with ice-cold PBS to remove floating cells and images were captured under an inverted microscope at 0 h, 24 h, and 48 h. The migration rate (%) = (0 h wound width − 24 h/48 h wound width)/0 h wound width × 100%. Each group had 3 parallel wells.

### 4.5. Infiltration Assay of MCF-7/ADR Cells by Three-Dimensional MCT Infiltration Model

The effect of UA on the infiltration ability of MCF-7/ADR cells was observed using a 3D MCT infiltration model. MCTs were obtained according to a method reported in the literature [43]. Briefly, 1.5% (*w*/*v*) agarose solution was prepared with serum-free RPMI-1640 medium, autoclaved for 30 min, and transferred to a 70 °C water bath on a clean bench while hot. Then, 50 μL of 1.5% (*w*/*v*) agarose solution was added to each well of a 96-well flat-bottomed plate and cooled for 30 min to fully solidify on a clean bench. Then, 200 μL of a cell suspension (5 × 10^4^ cells/mL) was added to each well of the processed 96-well plates, and the plate was placed in a 37 °C humidified culture incubator with 5% CO_2_ for 4 days. During this time, the door of incubator was carefully opened and closed to prevent the plates from being disturbed. The coculture of HUVECs and MCTs was based on the reported methods in the literature with slight modifications [44,45]. Briefly, 1.5 × 10^5^ HUVEC cells/well were seeded in 6-well plates and cultured for 72 h to completely cover the bottom of the well. Different drug-treated MCF-7/ADR MCTs were added to a 6-well plate fully covered with HUVECs at the bottom, and 6 MCTs were added to each well. The cells were co-cultured in an incubator, and the process of MCT infiltration into HUVECs was observed by light microscopy every day.

### 4.6. Molecular Docking

ODC is closely related to the development of tumors, so we adopted molecular docking to investigate the affinity of UA for ODC. The crystal structure of ODC was obtained from the Protein Data Bank (PDBID: 1D7K, resolution 2.1 Å) according to the published literature [21]. Further, this protein structure was processed with Autodock tools 1.5.6, included in the Autodock software; waters were removed and cofactor PLP was kept. The small molecules DFMO and UA were sketched and further processed to minimize the energy of the obtained 3D structures by the ChemDraw software. The 3D structures were also processed with Autodock tools 1.5.6 before docking. The computational docking of ligands and protein was carried out in the Autodock software, and the visualization was performed in the Pymol and Discovery Studio software.

### 4.7. Western Blot Assay

The molecular mechanism underlying the inhibitory activities of UA was further investigated by western blot analysis and related assay kits. Western blot analysis was carried out according to the reported literature [23]. Total proteins from MCF-7/ADR cells were extracted with RIPA lysis buffer containing 1% protease inhibitors and 1% phosphatase inhibitors. The protein concentration was determined with a BCA Protein Quantitative Kit (Nanjing Jiancheng Bioengineering Institute, Nanjing, China). The protein samples were added to 5× loading buffer and boiled for 15 min in a metal bath at 97 °C. Then, 10% sodium dodecyl sulfate–polyacrylamide gel electrophoresis (SDS-PAGE) was performed to separate the proteins. A PVDF membrane loaded with protein was blocked with 5% non-fat dry milk for 2 h at room temperature. Next, the membrane was incubated with primary antibodies against MMP-9, VEGF, ODC, GAPDH, and total and phosphorylated Erk1/2 at 4 °C overnight. Subsequently, the membrane was incubated with secondary antibody for 2 h at 37 °C. Intensive TBST (Tris-buffered saline containing 0.1% Tween 20) washing was performed after each incubation. Finally, the membrane was exposed to enhanced chemiluminescence (ECL) reagents and photographed using the Tanon Imaging system (Shanghai, China).

The activity of MMP-9 in the culture medium was determined by the assay kit according to the manufacturer’s protocol. Briefly, the culture medium was centrifuged at 1000× *g* for 20 min and the supernatant was collected to measure MMP-9 activity following the manufacturer’s instructions.

### 4.8. Quantification of Metabolites Involved into Polyamine Metabolism

Quantitative analysis was measured by UPLC–MS/MS. MCF-7/ADR and MCF-7 cells at a density of 2 × 10^5^ cells/well were seeded in 6-well plates. After overnight adhesion, the drugs (4 μM Dox, 2 μM UA, 16 μM UA, 4 μM Dox + 2 μM UA, and 4 μM Dox + 16 μM UA) were added to corresponding wells; each group contained 6 parallel wells. After 48 h, 300 μL of the cell culture medium was harvested in a 1.5 mL centrifuge tube and the remaining culture medium was discarded. The cells were washed twice with ice-cold PBS, trypsinized, washed once with ice-cold PBS, and centrifuged at 1000 rpm to obtain a cell pellet, to which 150 μL of ultrapure water was added. After three freeze–thaw cycles (frozen at −80 °C, thawed at 37 °C), the supernatant was obtained by centrifugation. Then, 20 μL of supernatant was used to determine the protein concentration with the Coomassie Protein Assay Reagent Kit (Nanjing Jiancheng Bioengineering Institute, Nanjing, China) according to the manufacturer’s protocol. The other aliquots were used for the extraction of metabolites. The extraction method of metabolites associated with polyamine metabolic pathways was adopted according to the reported literature [46]. The dried samples were resuspended in 80 μL and 100 μL of the initial mobile phase containing 1 μM 1,6-hexanediamine (internal standard), respectively, for the cell and culture medium sample and measured by UPLC–MS/MS.

### 4.9. Intracellular Accumulation of Dox in MCF-7/ADR MCTs

The effect of UA on the intracellular levels of Dox in MCF-7/ADR MCTs was analyzed by flow cytometry. MCTs in the 96-well plate were obtained using the methods of Section 4.5. After 4 days, 100 μL of culture medium was aspirated for each well, and another 100 μL of fresh culture medium containing 20 μM Dox, 20 μM Dox in combination with 4 μM UA, 16 μM UA, or 32 μM UA was added, respectively. The spheroids with the same diameter and volume were used for the assay; each group contained 12 parallel wells. After 48 h of culture, four MCTs in each group were combined, centrifuged in 4 °C PBS, and trypsinized for 10 min to allow full dispersion into a single-cell suspension, and the culture medium was then added to inactivate trypsin. The cell pellets were washed twice with 4 °C PBS. The fluorescence intensity of intracellular Dox was determined by an Accuri^TM^ C6 Plus flow cytometer (BD Biosciences, USA). Traditional 2D monolayer-cultured cells treated with 2 μM UA + 10 μM Dox, 8 μM UA + 10 μM Dox, and 16 μM UA + 10 μM Dox for 48 h were used as the control group, and sample treatment was the same as that of MCTs. Each measurement was performed on at least 10,000 cells. The cell population was selected from the scatter plot. Dox has a maximum emission wavelength of 591 nm at the excitation wavelength of 488 nm, and the amount of Dox was recorded as the mean fluorescence intensity in the PE channel. The data were analyzed using the FlowJo software (Treestar, Ashland, OR, USA).

### 4.10. UPLC-MS/MS Conditions

Instrumentation: Waters ACQUITY UPLC (Waters Corp., Milford, MA, USA); Xevo triple quadrupole mass spectrometer equipped with an electrospray ion source.

Chromatographic conditions: ACQUITY UPLC BEH amide column (100 mm × 2.1 mm, 1.7 μm) was used. The column temperature was 37 °C. The mobile phase consisted of acetonitrile (A) with 0.1% (*v*/*v*) formic acid and water with 0.1% (*v*/*v*) formic acid (B) and the gradient elution was as follows: 0–2 min, 99–64% A; 2–6 min, 64% A; 6–7 min, 64–60% A; 7–12 min, 60% A; 12–13 min, 60–99% A, 13–17 min, 99% A. Flow rate was 0.4 mL/min, the temperature of autosampler was 4 °C, and the injection volume was 10 μL.

Mass spectrometry conditions: multiple reaction monitoring (MRM) in positive ion mode was used, capillary voltage was 2.8 kV, ion source temperature was 150 °C, desolvation gas temperature was 350 °C, desolvation gas flow rate was 600 L/h, and cone gas flow rate was 50 L/h. The detailed MRM parameters for the analytes are shown in Table S1.

### 4.11. Statistical Analysis

Statistical data analysis was performed by ordinary one-way ANOVA analysis and Tukey’s multiple comparison test using the software OriginPro 8.5.0 (OriginLab, Northampton, MA, USA). Data were presented as the mean ± standard deviation from at least three replicates.

## 5. Conclusions

In this study, we investigated the effects of UA on the invasion and metastasis ability of MCF-7/ADR cells. The obtained results indicated that UA could reduce the adhesion and infiltration of MCF-7/ADR cells to HUVECs and the migration ability of MCF-7/ADR cells. UA had a higher affinity for the active residues in ODC compared to the known inhibitor DFMO. Furthermore, UA could inhibit the expression of ODC and VEGF, the phosphorylation of Erk1/2, the activity of MMP-9, and polyamine metabolism, eventually resulting in the suppression of metastasis in MCF-7/ADR cells. Moreover, the effects of UA combined with Dox were better than that of Dox alone; that is, UA could attenuate the enhanced metastasis ability induced by chemotherapy. Furthermore, UA could slightly increase the intracellular accumulation of Dox in MCF-7/ADR MCTs. It may also be one of the reasons for the significant inhibitory effect of UA combined with Dox. According to the above obtained results, we deduced that UA could be a potential clinical drug candidate to inhibit the metastasis of drug-resistant tumor cells and that further in vivo research is needed to explore the clinical value of UA.

## Figures and Tables

**Table 1:** Dox accumulation in 2D and 3D cells incubated with different concentrations of UA.

Cell Type	Fold Change ^1^		
	+2 μM UA+ 10 μM Dox	+8 μM UA+ 10 μM Dox	+16 μM UA+ 10 μM Dox
2D cells	1.15 *	1.12 **	1.06
3D cells	0.86	1.01	1.37

^1^ The group treated with UA combined with Dox versus the group treated with Dox alone, * *p* < 0.05, ** *p* < 0.01.

## Data Availability

The data presented in this study are available in this article and Supplementary Materials.

## Supplementary Materials

The following supporting information can be available online, Table S1: Multiple reaction monitoring (MRM) parameters for ten analytes; Figure S1 Original western blots images of ODC (*n* = 3), Figure S2: Original western blots images of Erk1/2 (*n* = 3), Figure S3: Original western blots images of P-Erk1/2 (*n* = 3), Figure S4: Original western blots images of VEGF (*n* = 3), Figure S5: Original western blots images of MMP-9 (*n* = 3), Figure S6: Original western blots images of GAPDH (*n* = 3).
