# Supplementary material for: Inhibitory Effect of Ursolic Acid on the Migration and Invasion of Doxorubicin-Resistant Breast Cancer
Source: Molecules. 2022 Feb 14;27(4):1282. doi: 10.3390/molecules27041282 (PMC8879026; doi:10.3390/molecules27041282)
Supplement: Supplementary file 1 [file molecules-27-01282-s001.zip › molecules-1554129-supplementary.pdf]

Inhibitory Effect of ursolic acid against the migration and invasion of Doxorubicin-resistant breast cancer

Li Zong[1, †], Guorong Cheng[1, †], Jingwu Zhao[1],Xiaoyu Zhuang[2*], Zhong Zheng[1], Zhiqiang Liu[1], Fengrui Song[1, *]

[1] State Key Laboratory of Electroanalytical Chemistry, Jilin Province Key Laboratory of Chinese Medicine Chemistry and Mass Spectrometry, Changchun Institute of Applied Chemistry, Chinese Academy of Sciences, Changchun 130022, China; zongliciac@126.com (L.Z.); mslab26@ciac.ac.cn (G.C.); mslab21@ciac.ac.cn (Z.P.); zhengzh@ciac.ac.cn (Z.Z.); liuzq@ciac.ac.cn (Z.L.)

[2] Experiment Center for Science and Technology, Shanghai University of Traditional Chinese Medicine, Shanghai, 201203, China;

[†] These authors contributed equally to this work.

* Correspondence: xyzhuang59@foxmail.com (X.Z.) and songfr@ciac.ac.cn (F.S.)

Table S1. Multiple reaction monitoring (MRM) parameters for ten analytes.

Analytes	MRM transition (m/z)	Cone voltage (V)	Collision energy (eV)	Ion mode
Arg	175.1>70.1[a]	14	12	positive
	175.1>60.1	14	18	positive
Orn	133.1>70.1[a]	16	14	positive
	133.1>116.0	16	10	positive
Met	150.0>104.1[a]	20	10	positive
	150.0>133.03	20	8	positive
SAM	399.3>136.1[a]	16	22	positive
	399.3>250.1	16	14	positive
MTA	298.2>136.1[a]	18	18	positive
	298.2>119.1	18	40	positive
Put	89.2>72.1[a]	10	8	positive
Spd	146.1>112.1[a]	18	10	positive
	146.1>72.2	18	14	positive
Spm	203.2>84.1[a]	18	28	positive
	203.2>112.2	18	18	positive
Acetylspm	245.3>129.1[a]	18	14	positive
	245.3>100.1	18	18	positive
IS	117.0>100.1[a]	30	10	positive
	117.0>55.2	30	16	positive

[a]precursor/product ion pair was used for quantification of the analytes.

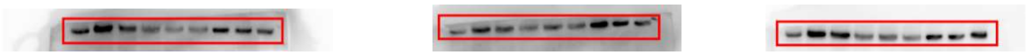

Figure S1. Original western blots imagesof ODC(n=3).

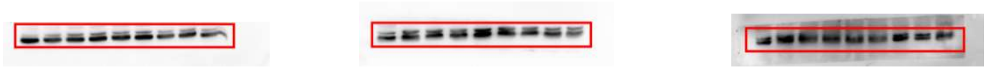

Figure S2. Original western blots imagesof Erk1/2(n=3).

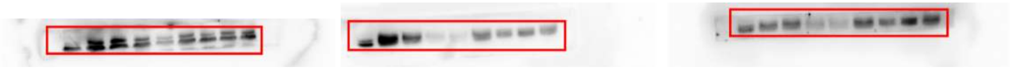

Figure S3. Original western blots imagesof P-Erk1/2 (n=3).

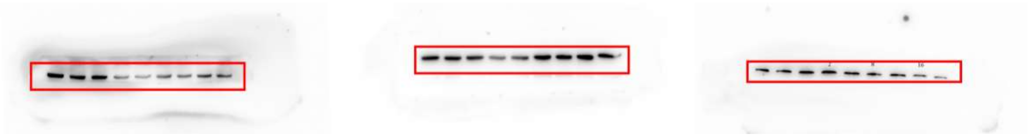

Figure S4. Original western blots imagesof VEGF (n=3).

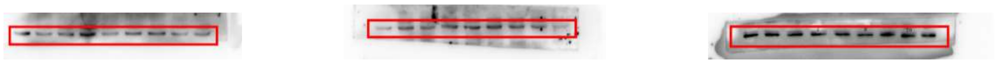

Figure S5. Original western blots imagesof MMP-9 (n=3).

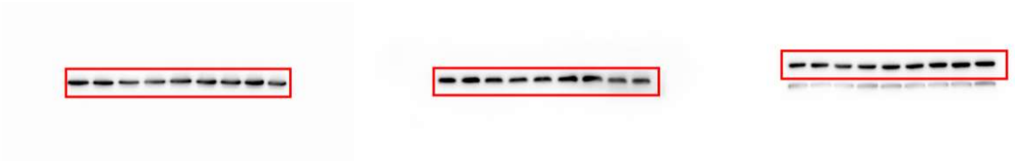

Figure S6. Original western blots imagesof GAPDH (n=3).
